# Global action to reduce HIV stigma and discrimination

**DOI:** 10.7448/IAS.16.3.18893

**Published:** 2013-11-13

**Authors:** Michel Sidibé, Eric P. Goosby

**Affiliations:** 1 Joint United Nations Programme on HIV/AIDS; 2 The President’s Emergency Plan for AIDS Relief US Department of State

This *JIAS* special issue examining HIV-related stigma and discrimination comes at a time when we see overwhelming evidence that global solidarity and shared responsibility are transforming the vision of an AIDS-free generation. A record of 10 million people living with HIV are now receiving treatment, far fewer people are dying from AIDS-related illnesses, 25 countries have reduced new forms of HIV infections by more than 50%, and new HIV treatment and prevention science promise yet more results. Yet, our work is far from over.

This is particularly true when it comes to fighting discrimination and stigma. The PEPFAR Blueprint, published last World AIDS Day, provides a roadmap for how we are partnering with countries to achieve an AIDS-free generation, and calls for an end to stigma and discrimination against people living with HIV and key populations.

HIV-related stigma and discrimination continues to endanger people living with the virus, and it still prevents millions of people from coming forward for testing and for prevention and treatment services. Some 50–60% of people living with HIV are unaware of their status. Many others choose to hide it. Communities most affected by the epidemic – sex workers, people who use drugs, men who have sex with men and transgender people –remain highly stigmatized. These individuals and their families are often unable to exercise their right to health, non-discrimination and freedom from violence.

At the 2011 UN High Level Meeting on HIV/AIDS, Member States committed to the goal of reducing stigma, discrimination and violence related to HIV. It is time to redouble our efforts. Countries must intensify their actions to build effective stigma-reduction programmes and policies; protective laws and protocols; and appropriate legal, social and policy frameworks that will eliminate stigma, discrimination and violence related to HIV. It is a global shared responsibility, and one that includes continued research into causes, manifestations and new metrics and monitoring approaches.

This issue of *JIAS* examines HIV-related stigma in a variety of contexts and settings and explores its impact on several populations, including medical students in Puerto Rico, church leaders in the United States, men who have sex with men in Swaziland and healthcare workers around the world.

Ending HIV-related stigma and discrimination will take considerable investment of time and resources, but our commitment is steadfast, and we are grateful to those who keep showing us how to do it better. The rights of all people living with or affected by HIV must be protected. It is that simple.


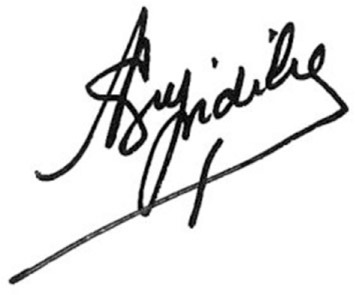
Michel Sidibé Executive Director Joint United Nations Programme on HIV/AIDS
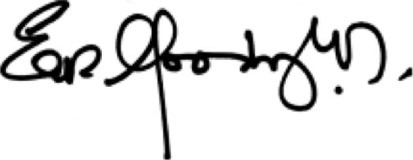
Eric P. Goosby, MD, RADM Ambassador at Large and US Global AIDS Coordinator The President's Emergency Plan for AIDS Relief US Department of State

